# Blood-Based Genomic Alteration Signature for Predicting Progression-Free Survival in *De Novo* Metastatic Hormone-Sensitive Prostate Cancer: A Real-World Study

**DOI:** 10.1158/2767-9764.CRC-25-0384

**Published:** 2025-12-01

**Authors:** Ruiliang Wang, Jing Xu, Chenyi Zhu, Wujianhong Liu, Chengqi Jin, Wentao Luo, Tingting Zhao, Changcheng Guo, Wei Chen, Bin Yang

**Affiliations:** 1 Department of Urology, Zhongshan Hospital, Fudan University, Shanghai, China.; 2 Department of Urology, Shanghai Tenth People’s Hospital, Tongji University School of Medicine, Shanghai, China.; 3 Urologic Cancer Institute, Tongji University School of Medicine, Shanghai, China.; 4 Department of Pathology, Shanghai Tenth People’s Hospital, Tongji University School of Medicine, Shanghai, China.; 5 School of Life Sciences and Technology, Tongji University, Shanghai, China.; 6 Research Institute, GloriousMed Clinical Laboratory, Shanghai, China.

## Abstract

Metastatic hormone-sensitive prostate cancer (mHSPC) often progresses to castration-resistant prostate cancer despite current therapies, necessitating the use of reliable biomarkers. This study aimed to develop a novel model for predicting progression-free survival (PFS) using ctDNA sequencing. We analyzed 127 patients with mHSPC and compared ctDNA mutations with those in matched primary tumor tissues. A four-gene signature (TRPC: *TP53*, *RB1*, *PTEN*, and *CDK12*) was identified, forming the basis of the blood-based TRPC (b.TRPC) model. The b.TRPC model demonstrated high specificity and sensitivity in predicting PFS, outperforming ctDNA markers. Internal and external validation confirmed that b.TRPC is an independent prognostic factor with superior predictive performance for 0.5-, 1-, and 2-year PFS. The model also showed significant clinical relevance, with b.TRPC-positive patients exhibiting shorter survival times under androgen deprivation therapy and doublet therapy, although this disparity diminished with triplet therapy. These findings highlight the potential of ctDNA-based gene mutation analysis to guide personalized treatment strategies for mHSPC, offering a noninvasive alternative to tissue-based analyses and improving prognostic accuracy.

**Significance:**

This study identified a novel noninvasive blood-based biomarker model (b.TRPC) using ctDNA to predict PFS in mHSPC. Analyzing *TP53*, *RB1*, *PTEN*, and *CDK12* alterations, it outperformed traditional ctDNA% markers. Findings highlight ctDNA-based biomarkers’ potential to guide personalized treatment, bridging real-world and trial data to aid mHSPC management.

## Introduction

Metastatic hormone-sensitive prostate cancer (mHSPC) represents a critical phase of disease progression, in which standard therapies combining androgen deprivation therapy (ADT) with novel hormonal therapies, chemotherapy, or radiotherapy fail to prevent progression to castration-resistant prostate cancer (CRPC) in most patients (1–8). Despite initial therapeutic responses, 60% to 70% of patients develop CRPC within 3 years, leading to poor survival outcomes (9, 10). This clinical urgency underscores the need for reliable biomarkers to predict disease progression and guide personalized therapeutic strategies.

Current predictive biomarkers rely heavily on clinical parameters, such as tumor burden and PSA kinetics, as well as tissue-based genomic profiling, including homologous recombination repair mutations (11–16). However, tissue biopsies suffer from spatial heterogeneity, sampling bias, and impracticality of serial monitoring (17–19). Liquid biopsy, particularly ctDNA, offers a dynamic alternative for capturing systemic tumor heterogeneity (20–22). Recent studies have highlighted ctDNA as a reliable predictive tool, serving as an alternative to tissue biopsy for identifying patients with metastatic CRPC who may benefit from olaparib, with 81% positive percentage agreement and 92% negative percentage agreement for detecting *BRCA/ATM* mutations compared with tumor tissue in the PROfound study (20, 23). This complements tissue testing to guide therapy while aiding risk stratification and biomarker analysis in metastatic CRPC (20, 24, 25). In mHSPC, ADT-induced ctDNA suppression and incomplete metastatic clone representation due to primary polyclonality limit ctDNA as a tissue substitute (26–28). However, independent ctDNA-based predictive models are needed for therapeutic guidance. Studies have shown that somatic alterations in PTEN, cell-cycle regulators, and chromatin modulators detected in ctDNA are associated with shorter progression-free survival (PFS) and overall survival (OS; ref. 29). These findings highlight the critical prognostic impact of ctDNA gene changes, supporting ctDNA-driven modeling despite the challenges (30, 31).

This study aimed to establish a ctDNA-driven predictive model that integrated longitudinal genomic and clinical data. We propose a minimally invasive framework to optimize treatment selection and predict survival outcomes in real time by identifying ctDNA-specific alterations and validating their prognostic superiority over established ctDNA- and tissue-based markers. This approach addressed the unmet need for dynamic risk stratification in mHSPC management.

## Materials and Methods

### Patients and samples

This training set analyzed data from 127 consecutive patients with mHSPC who were treated at the Shanghai Tenth People’s Hospital (Shanghai, China) between January 2018 and August 2021. Tumor tissues were obtained through biopsy or surgery, and ctDNA from peripheral blood was collected within 1 week before biopsy or surgery. The external validation set was collected from public databases and published literature (31).

This study was approved by the Ethics Committee of the Shanghai Tenth People’s Hospital (approval number: SHSY-IEC-5.0/22K50/P01). All patients provided written informed consent for their anonymized medical data to be analyzed and published for research purposes at the time of treatment. The study was conducted in accordance with the ethical principles outlined in the Declaration of Helsinki, Council for International Organizations of Medical Sciences guidelines, Belmont Report, and US Common Rule.

### DNA sequencing and bioinformatics

**Figure 1.**
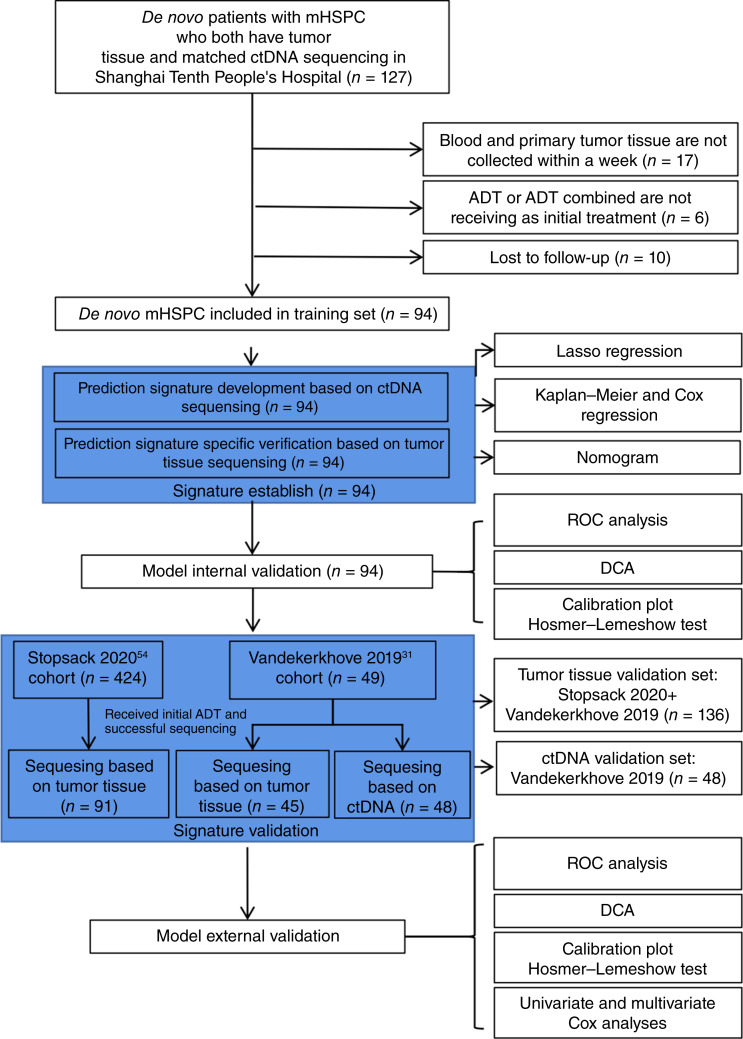
Study design and patient cohort selection.

Tumor tissues and blood samples were collected and processed according to the methodology described in our previous studies (25, 32, 33). Targeted next-generation sequencing was performed at the GloriousMed Clinical Laboratory (Shanghai, China), utilizing a 620-gene panel on 72 tumor tissue samples and 79 plasma samples, whereas an additional 17 tumor samples and 15 plasma samples underwent targeted sequencing with a 66-gene panel. Furthermore, whole-exome sequencing was conducted on five tumor tissue samples (Fig. 1A; Supplementary Fig. S1). Germline mutations, somatic mutations, and copy number variants were identified using the Trimmomatic and Burrows-Wheeler transformation tools (34–36). A total of 94 patients with mHSPC were included in the training cohort. Among the 127 initially enrolled, 17 patients were excluded because blood and primary tumor tissue were not collected within a week, six were excluded because they did not receive ADT or ADT combination as the initial treatment, and 10 patients were lost to follow-up.

### Establishment of prediction models

Lasso Cox regression analysis was initially performed to identify the optimal combination of genes for prognostic risk prediction. To further refine these genes, a log-rank test was performed to evaluate the association between each candidate gene and patient survival outcomes. Genes that exhibited significant *P* values in the log-rank test were selected as candidate genes, indicating a meaningful association with survival outcomes. Finally, the model was simplified by categorizing the patients into distinct risk groups based on the number of gene alterations. Patients were classified as having either one or two or more gene alterations. This categorization aimed to create more streamlined and clinically relevant risk stratification, facilitating the interpretation and application of the prognostic model in a practical setting.

### Survival outcomes

Patients were closely followed up every 3 to 12 weeks after the initiation of therapy. At follow-up visits, tumor biomarkers, such as PSA, testosterone, liver and kidney function, complete blood count, electrolytes, imaging, and patient symptoms, were detected, and progressive lesions were assessed using multimodal imaging. Disease stage and regression or progression were assessed based on the Prostate Cancer Working Group 3.0 criteria and RECIST 1.1 criteria. Patients were followed up until death or September 30, 2021. PFS was defined as the time from the initiation of ADT to confirmed biochemical progression, clinical or radiographic progression, or death (37, 38). Deaths without documented progression were counted as events rather than censored.

### Development of the nomogram

A nomogram was developed based on the factors that were significant in the final multivariate Cox regression analyses. ROC, concordance index (C-index), and calibration plots were used to assess the performance of the nomogram. Nomogram, C-index, and calibration plots were generated using the “Rms” R package v6.1. Furthermore, decision curve analysis (DCA) was used to ascertain the net benefits of the nomogram and other crucial prognostic factors.

### Statistical analysis

Data were analyzed using GraphPad Prism v8 (GraphPad Software), SPSS 25.0, (IBM) or R v4.4.0 (https://www.r-project.org/). Results associated with two-sided *P* < 0.05 were considered statistically significant unless otherwise noted. PFS was analyzed using the Kaplan–Meier method, and the curves were compared using the log-rank test. Risk factors for poor survival were identified by conducting univariate analysis, the significant factors from which (based on *P* < 0.01) were entered into a multivariate Cox proportional hazard regression. The results were reported as HRs and 95% confidence intervals (CI).

## Results

### Patient characteristics

A total of 127 patients with mHSPC were included in this study (Fig. 1), of whom 94 were enrolled from the Shanghai TenthPeople’s Hospital. Baseline clinical characteristics are summarized in Table 1. Fifty-six patients (58.7%) had a Gleason score >8 at diagnosis. In total, 26 patients reached the PFS endpoint. The median PFS for the entire cohort was 12.55 months (IQR, 9.1–24.3 months). Among the 10 patients for whom death was the PFS event, the median follow-up was 8.63 months (IQR, 5.61–12.19 months), whereas the 16 patients with biochemical, clinical, or radiographic progression had a median follow-up of 13.2 months (IQR, 9.20–26.4 months). The median ctDNA fraction was 4.7% (IQR, 2.1%–10.8%).

**Table 1.** Clinical characteristics at diagnosis with mHSPC.

Characteristics	Result
PSA level at diagnosis, *n* (%)	​
≤20 ng/mL	15 (16%)
20–100 ng/mL	33 (35.1%)
>100 ng/mL	46 (48.9%)
Gleason score, *n* (%)	​
≤8	36 (38.3%)
>8	56 (59.6%)
Unknown	2 (2.1%)
Disease volume, *n* (%)	​
Low	57 (60.6%)
High	37 (39.4%)
Treatment, *n* (%)	​
ADT only	17 (18.1%)
ADT + AR-targeted therapy^a^	27 (28.7%)
ADT + docetaxel	42 (44.7%)
ADT + docetaxel + AR-targeted therapy^a^	8 (8.5%)
ctDNA, median (IQR)	4.673 (2.0883, 10.792)
Metastatic extent of disease at diagnosis, *n* (%)	​
Nonregional lymph node only	23 (24.5%)
Bone (≥4)	27 (28.7%)
Bone (<4)	34 (36.2%)
Viscera	10 (10.6%)
PFS, median, month (IQR)	12.55 (9.1, 24.317)

Abbreviation: AR, androgen receptor.

a Abiraterone acetate, enzalutamide, or apalutamide.

### Construction of the alternative genes signature

The results of the Lasso Cox regression analysis are shown in Supplementary Fig. S1. The optimal gene combination for prognostic risk prediction was selected based on the log λ validation obtained from the Lasso Cox model (Supplementary Fig. S1A and S1B). We found that the combination of the six genes yielded the best prediction performance, as indicated by the estimated C-index (Supplementary Fig. S1B). Specifically, Lasso Cox regression analysis identified six genes (*RB1*, *PTEN*, *CDK12*, *TP53*, *FANCA*, and *AR*) with nonzero coefficients, which formed the gene signature set (Supplementary Fig. S1C). Each gene in the signature was calculated for each patient. Kaplan–Meier analysis further demonstrated that *TP53*, *RB1*, *PTEN*, and *CDK12* played a significant role (*P* < 0.05) in survival outcomes (Supplementary Fig. S1D–S1G). To assess whether the TRPC model functions independently in blood samples [blood-based TRPC (b.TRPC)], we developed a TRPC model for tissue samples [tissue-based TRPC (t.TRPC)]. After performing both univariate and multivariate analyses, we found that b.TRPC was an independent prognostic factor among all clinical variables (Supplementary Table S1).

### Genomic alterations in tumor tissues and ctDNA

**Figure 2.**
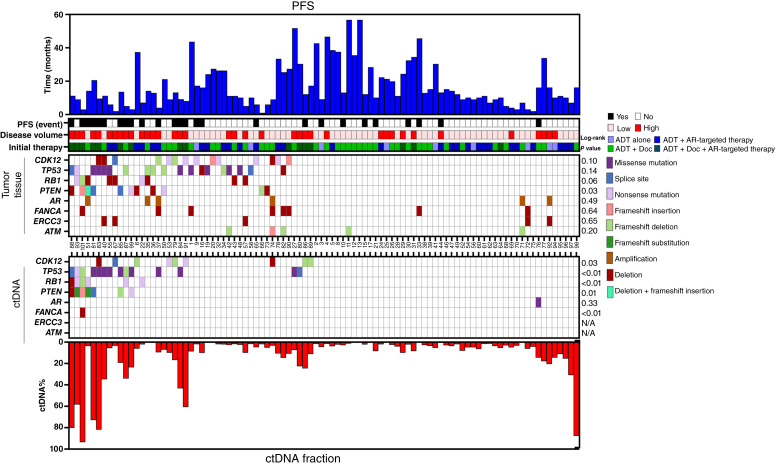
Genomic landscape and concordance of alterations in tumor tissue and ctDNA. AR, androgen receptor; Doc, docetaxel; N/A, not applicable.

In the landscape, genes with an alteration frequency exceeding 5% in either tumor tissue or ctDNA are shown. These include *TP53*, *RB1*, *PTEN*, *CDK12*, *AR*, *FANCA*, *ERCC3*, and *ATM* (Fig. 2). In tumor tissues, the alteration frequencies of the four key genes were as follows: *TP53* (21.2%), *RB1* (9.5%), *PTEN* (11.7%), and *CDK12* (13.8%). The ctDNA alteration frequencies were *TP53* (17.1%), *RB1* (11.7%), *PTEN* (10.6%), and *CDK12* (10.6%). For these four genes, a total of 22 genes (8 for *TP53*, 3 for *RB1*, 4 for *PTEN*, and 7 for *CDK12*) were found to be the same altered in both tumor tissues and ctDNA, yielding a concordance rate of 40.7%. Additionally, 32 alterations (11 for *TP53*, 6 for *RB1*, 7 for *PTEN*, and 8 for *CDK12*) were exclusive to tumor tissues, whereas 13 alterations (6 for *TP53*, 3 for *RB1*, 3 for *PTEN*, and 1 for *CDK12*) were unique to ctDNA. Further details about clinical data and specific gene alterations are provided in Supplementary Tables S2 and S3.

### Internal and external validation of the b.TRPC model

**Figure 3.**
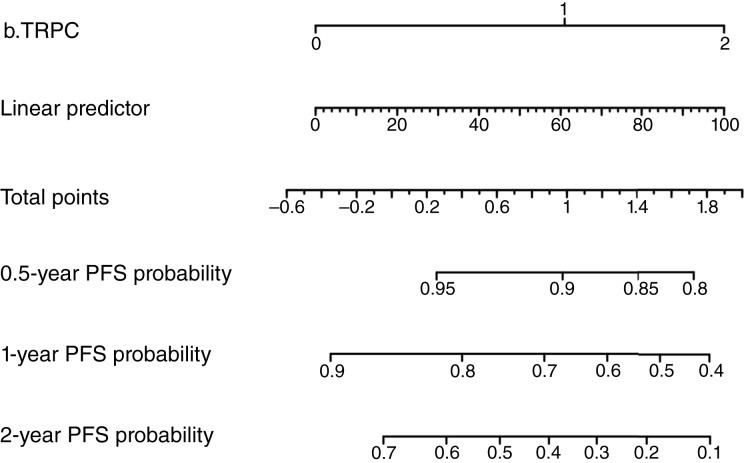
Nomogram for predicting PFS based on b.TRPC score.

To validate the performance of the b.TRPC model, an independent multivariate analysis using blood samples was conducted, which confirmed that b.TRPC was an independent prognostic factor (Supplementary Table S4). Based on these analyses, a nomogram was constructed to predict the 0.5-, 1-, and 2-year PFS rates (Fig. 3). The ctDNA% marker was included as a comparator.

**Figure 4.**
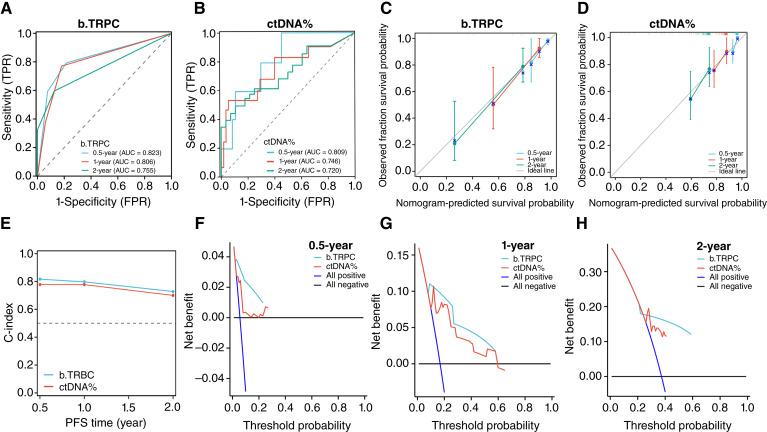
Internal validation of the b.TRPC model and comparison with ctDNA%. **A** and **B,** ROC curves comparing the predictive performance of b.TRPC and ctDNA% for 0.5-, 1-, and 2-year CRPC-free survival. **C** and **D,** Calibration plots showing the agreement between predicted and observed survival probabilities for b.TRPC and ctDNA%. **E,** C-index values demonstrating the superior prognostic accuracy of b.TRPC compared with ctDNA%. **F–H,** DCA illustrating the net clinical benefit of b.TRPC across different time points. FPR, false positive rate; TPR, true positive rate.

ROC curve analysis showed that the AUC values for b.TRPC were 0.823, 0.806, and 0.755 for the 0.5-, 1-, and 2-year predictions, whereas ctDNA% showed AUC values of 0.809, 0.746, and 0.720 (Fig. 4A and B), respectively.

Calibration plots demonstrated close agreement between the predicted and observed survival probabilities for b.TRPC and ctDNA% across all time points, indicating good calibration (Fig. 4C and D).

The C-index for b.TRPC was 0.749 (95% CI, 0.704–0.795), which was higher than that for ctDNA% at 0.727 (95% CI, 0.676–0.779; Fig. 4E).

In DCA, the net benefit of b.TRPC exceeded that of ctDNA% across threshold probabilities at 0.5, 1, and 2 years, indicating greater clinical applicability (Fig. 4F–H).

**Figure 5.**
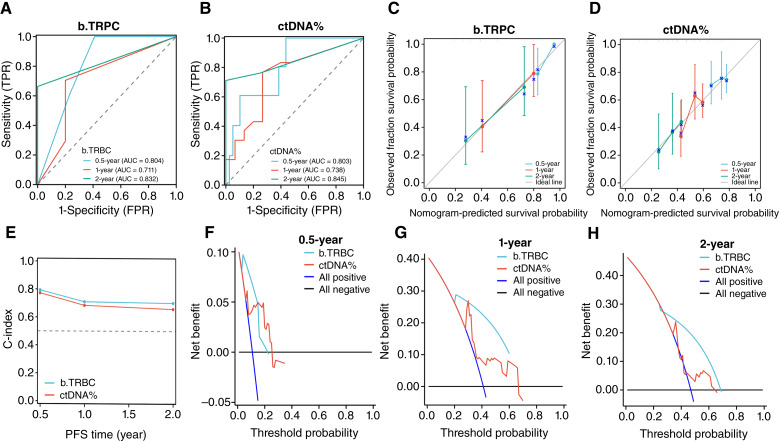
External validation of the b.TRPC model in an independent dataset. **A** and **B,** ROC curves assessing the predictive performance of b.TRPC for 0.5-, 1-, and 2-year CRPC-free survival in the external validation cohort. **C** and **D,** Calibration plots confirming the reliability of the b.TRPC model across all time points. **E,** Comparison of C-index values for b.TRPC and ctDNA% in the external dataset. **F–H,** DCA demonstrating the clinical utility of b.TRPC for risk stratification in an independent patient cohort. FPR, false positive rate; TPR, true positive rate.

In the validation dataset, b.TRPC maintained AUC values of 0.804, 0.711, and 0.832 for the 0.5-, 1-, and 2-year predictions, whereas ctDNA% exhibited AUC values of 0.803, 0.738, and 0.845 (Fig. 5A and B), respectively. The calibration plots again showed strong alignment with the observed values (Fig. 5C and D), and the C-index for b.TRPC was 0.729 (95% CI, 0.674–0.785) compared with 0.718 (95% CI, 0.665–0.772) for ctDNA% (Fig. 5E). DCA confirmed that b.TRPC offered a higher net benefit than ctDNA% across all examined time points (Fig. 5F–H).

### Verifying TRPC model conditional independencies on ctDNA

To validate whether the TRPC gene combination (TRPC) acts independently of ctDNA, we conducted analyses using matched tumor tissue sequencing data (Supplementary Table S5). Multivariate analysis of the tumor tissue identified disease volume and the t.TRPC model as independent prognostic factors. We constructed a combined predictive model of t.TRPC and disease volume (t.TRPC + disease volume) as a nomogram (Supplementary Fig. S2) and included this combined model along with t.TRPC and disease volume as individual variables in subsequent comparative analyses.

The ROC analysis revealed that the AUC values for t.TRPC at 0.5, 1, and 2 years were 0.785, 0.760, and 0.722, respectively. For disease volume, the AUC values at 0.5, 1, and 2 years were 0.719, 0.646, and 0.677, respectively. The combined model (t.TRPC + disease volume) achieved higher AUCs of 0.895, 0.795, and 0.749 for the 0.5-, 1-, and 2-year predictions, respectively (Supplementary Fig. S3A–S3C). The calibration curves demonstrated good predictive performance for t.TRPC, disease volume, and t.TRPC + disease volume across 0.5, 1, and 2 years (Supplementary Fig. S3D–S3F).

The C-index analysis further supported the predictive superiority of the combined model. The C-index for t.TRPC was 0.681 (95% CI, 0.637–0.726), for t.TRPC + disease volume was 0.734 (95% CI, 0.678–0.790), and for disease volume alone was 0.647 (95% CI, 0.597–0.696; Supplementary Fig. S3G). In the DCA at 0.5, 1, and 2 years, Supplementary Fig. S3H–S3J illustrates the comparative DCA curves of the tumor tissue–based models (t.TRPC + disease volume, t.TRPC, and disease volume) against the b.TRPC model. The results indicated that, compared with low-risk patients, b.TRPC and t.TRPC + disease volume demonstrated similar or highest net benefits, with b.TRPC significantly outperforming both t.TRPC and disease volume.

In the validation set, ROC analysis showed that the AUC values for t.TRPC were 0.517, 0.618, and 0.672 for the 0.5-, 1-, and 2-year predictions, respectively. Disease volume showed AUC values of 0.511, 0.635, and 0.702, whereas t.TRPC + disease volume achieved AUCs of 0.521, 0.647, and 0.704 at 0.5, 1, and 2 years, respectively (Supplementary Fig. S4A–S4C). Calibration curve analysis indicated that the t.TRPC + disease volume model was closest to the ideal line, suggesting superior calibration (Supplementary Fig. S4D–S4F).

The C-index analysis in the validation cohort also highlighted the advantage of the combined model, with t.TRPC + disease volume achieving a C-index of 0.734 (95% CI, 0.678–0.790), compared with t.TRPC of 0.550 (95% CI, 0.516–0.585) and disease volume of 0.549 (95% CI, 0.518–0.581; Supplementary Fig. S4G). In DCA, all three tissue-based models showed lower net benefits than the “all positive” and “all negative” lines at the 0.5-year mark. However, at 1 and 2 years, the t.TRPC + disease volume model provided the highest predictive benefit, demonstrating its potential as a more effective long-term predictor (Supplementary Fig. S4H–S4J).

### Clinical significance of TRPC

**Figure 6.**
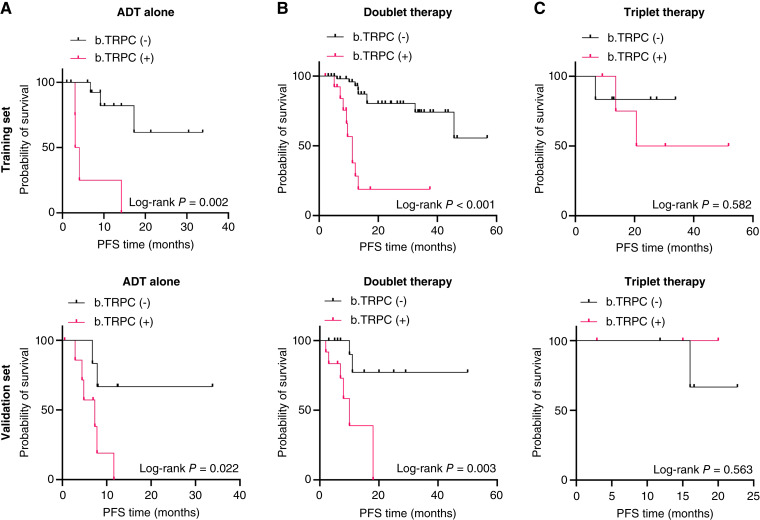
Clinical significance of b.TRPC in patients undergoing different therapeutic regimens. **A,** Kaplan–Meier curves of progression-free survival (PFS) in b.TRPC-positive vs. b.TRPC-negative patients receiving ADT monotherapy. **B,** PFS outcomes in b.TRPC-positive and b.TRPC-negative patients treated with doublet therapy. **C,** Kaplan–Meier analysis of PFS in b.TRPC-positive and b.TRPC-negative patients administered triplet therapy. Training set (top); validation set (bottom).

In patients receiving ADT alone, those who tested positive for b.TRPC exhibited significantly shorter PFS than b.TRPC-negative patients in both the training and validation sets (Fig. 6A and B). Similarly, among patients undergoing doublet therapy (ADT combined with additional treatment), b.TRPC-positive patients showed worse survival outcomes than b.TRPC-negative patients in both the training and validation sets (Fig. 6C and D). However, in patients receiving triplet therapy, there was no statistically significant difference in survival between the b.TRPC-positive and b.TRPC-negative groups (Fig. 6E and F).

## Discussion

Our study analyzed the prognostic significance of a ctDNA-based prognostic model in patients with mHSPC. Our findings demonstrate that the b.TRPC model functions as an independent predictor of disease progression and survival, offering a noninvasive alternative to tissue-based analyses. This blood-based model could potentially streamline the prognosis for patients with mHSPC, as ctDNA provides a snapshot of tumor-specific genomic alterations, making it a convenient biomarker in clinical settings.

Our results demonstrated that the b.TRPC model, a blood-based prognostic model detecting alterations in four genes, significantly outperformed the t.TRPC model that identified the same four gene alterations in tumor tissue. Blood-based prognostic models offer several advantages over tissue-based ones. Blood sample collection is noninvasive, allowing for repeated testing throughout the disease course, which aids in the real-time monitoring of tumor progression and treatment response. ctDNA provides a comprehensive snapshot of tumor-specific genetic alterations, overcoming the limitations of tissue sampling such as tissue heterogeneity and biopsy site variability. The b.TRPC model, which focuses on specific tumor-related genes, enhances detection sensitivity and accuracy, even in cases of low tumor burden, and reduces the number of false positives from nontumor DNA. Moreover, it is cost-effective, easier to implement, and suitable for large-scale clinical use. Overall, blood-based models offer a more convenient, accurate, and comprehensive approach for monitoring and predicting outcomes in mHSPC, advancing precision medicine in prostate cancer treatment.

Our findings indicate that the b.TRPC model outperforms ctDNA% as a biomarker, offering superior predictive and monitoring capabilities for mHSPC outcomes. ctDNA% is a valuable predictive and monitoring biomarker for prostate cancer, particularly in CRPC (27, 39). Elevated baseline ctDNA% correlates with poorer PFS and OS in CRPC, whereas dynamic changes during treatment indicate therapeutic response and resistance (31, 40, 41). However, some issues remain with HSPC. Notably, ADT significantly decreases ctDNA abundance in mHSPC, which affects its detection (26, 31). Moreover, detection sensitivity is limited, especially for low-level ctDNA and some common copy-number alterations (27). Consequently, in mHSPC, ctDNA-based molecular stratification should be developed to enhance personalized treatment strategies by guiding therapeutic decisions, especially when traditional biomarkers such as PSA are insufficient (42). b.TRPC, the targeted approach, not only enhances clinical relevance but also increases sensitivity in detecting low ctDNA levels. In addition, the model is cost-effective and less technically demanding, making it more suitable for large-scale clinical applications. Focusing on tumor-specific genes can also reduce false positives caused by nontumor DNA sources, such as inflammation or normal cell turnover (24). Moreover, dynamic changes in specific gene alterations could provide deeper insights into tumor progression and treatment resistance, surpassing the limitations of ctDNA% as a general marker of tumor burden (43). This simplified, biologically focused model shows promise in improving cancer detection and monitoring.

Our results demonstrate that the b.TRPC model, a blood-based prognostic tool that detects alterations in four genes, significantly outperforms the t.TRPC model, which identifies the same gene alterations in the tumor tissue. Blood-based models provide several advantages over traditional tissue-sampling methods. Initially, the noninvasive nature of blood sample collection allows for repeated testing throughout the disease course, facilitating real-time monitoring of tumor progression and therapeutic response (44, 45). Moreover, ctDNA serves as a comprehensive biomarker for capturing tumor-specific genetic alterations and overcoming the limitations inherent in tissue sampling, such as tumor heterogeneity and biopsy site variability (30, 46). Notably, by focusing on key tumor-related genes, the b.TRPC model enhances detection sensitivity and accuracy, even in cases of low tumor burden, while minimizing false positives from nontumor DNA sources such as normal cell turnover or inflammation (47–50). Furthermore, the b.TRPC model was cost-effective and easier to implement, making it more suitable for large-scale clinical applications. Collectively, these attributes position blood-based models as a more convenient, accurate, and comprehensive approach for monitoring and predicting outcomes in mHSPC, offering new opportunities for precision medicine in prostate cancer treatment.

Our findings highlight the utility of the b.TRPC signature, comprising *TP53*, *RB1*, *PTEN*, and *CDK12* alterations detected in ctDNA, as a robust prognostic marker of survival outcomes in prostate cancer. This aligns with and extends prior evidence associating cumulative disruptions in *TP53*, *PTEN*, and *RB1* with aggressive disease phenotypes and diminished survival rates (51–53). These genomic alterations collectively underpin the critical mechanisms of tumor progression, including enhanced genomic instability, evasion of apoptosis, and resistance to therapeutic interventions. Intriguingly, their predictive value extends to a more rapid transition to CRPC, underscoring their pivotal role in shaping disease trajectories, particularly in early-stage mHSPC (54). The inclusion of *CDK12* mutations within this signature further reinforces its significance, given its established role in fostering defects in DNA damage repair pathways, which are increasingly recognized as therapeutic vulnerabilities (55–57). These insights not only deepen our understanding of the molecular mechanisms driving prostate cancer progression but also highlight potential avenues for precision medicine strategies, emphasizing the need for early, tailored interventions to mitigate therapy resistance and improve patient outcomes.

The clinical relevance of b.TRPC in treatment outcomes has been further underscored by recent studies. Among patients with prostate cancer undergoing ADT, those who were b.TRPC-positive exhibited significantly shorter survival than those who were b.TRPC-negative. This trend persisted in dual therapy settings (ADT combined with chemotherapy or antiandrogen therapy), in which b.TRPC-positive patients continued to show poorer survival outcomes. However, in triplet therapy settings, ADT combined with docetaxel and additional targeted agents, the survival disparities between b.TRPC-positive and b.TRPC-negative patients began to diminish. These observations are consistent with recent clinical trials, such as PEACE-1 and ARASENS, which demonstrated substantial improvements in OS and PFS in patients with high-burden, hormone-sensitive metastatic prostate cancer when treated with triplet therapy (6, 58). The potential for personalized treatment strategies based on b.TRPC status is supported by these findings, indicating that high-risk patients may benefit from more aggressive treatment approaches, particularly with the evolving standard of triplet therapy for HSPC (59). By integrating ADT, docetaxel, and antiandrogen therapies, such as abiraterone or enzalutamide, triplet therapy effectively mitigates some of the adverse prognostic factors associated with b.TRPC positivity, offering extended survival benefits for these high-risk patients (60).

In conclusion, our study provides novel insights into the prognostic utility of the b.TRPC model for mHSPC. Our results support the integration of ctDNA analysis with traditional tissue-based approaches to obtain a more comprehensive genomic profile. Future research should validate these findings in larger cohorts and explore advanced technologies, such as multi-omics, to enhance the sensitivity of ctDNA detection. Ultimately, combining blood-based biomarkers with targeted therapies could optimize treatment outcomes and offer a personalized approach for managing mHSPC.

## Supplementary Material

Supplementary Figure S1 Figure S1. Construction of the alternative genes signature for prognostic risk prediction.

Supplementary Figure S2 Figure S2. Nomogram for predicting progression-free survival (PFS) based on t.TRPC and disease volume score.

Supplementary Figure S3 Figure S3. Internal validation and clinical utility of the t.TRPC-based prognostic model.

Supplementary Figure S4 Figure S4. External validation and clinical applicability of the t.TRPC-based prognostic model in the validation cohort.

Supplementary Table S1 Analysis for b.TRPC as an Independent Prognostic Factor

Supplementary Table S2 Genomic Alterations in Tumor Tissue and ctDNA

Supplementary Table S3 Clinical Data Correlated with Genomic Alterations

Supplementary Table S4 Multivariate Analysis of Prognostic Factors Based on ctDNA

Supplementary Table S5 Multivariate Analysis of Prognostic Factors Based on Tumor Tissue

## Data Availability

The data generated in this study are not publicly available because of patient privacy but are available upon reasonable request in accordance with Chinese regulations on the management of human genomic resources policy. However, interested parties can obtain these data by making reasonable requests to the corresponding author. The remaining data generated in this study are available in the article and Supplementary Data.
