# Supplementary figures and images for: Blood-Based Genomic Alteration Signature for Predicting Progression-Free Survival in De Novo Metastatic Hormone-Sensitive Prostate Cancer: A Real-World Study
Source: Cancer Res Commun. 2025 Dec 1;5(12):2092–101. doi: 10.1158/2767-9764.CRC-25-0384 (PMC12665648; doi:10.1158/2767-9764.CRC-25-0384)

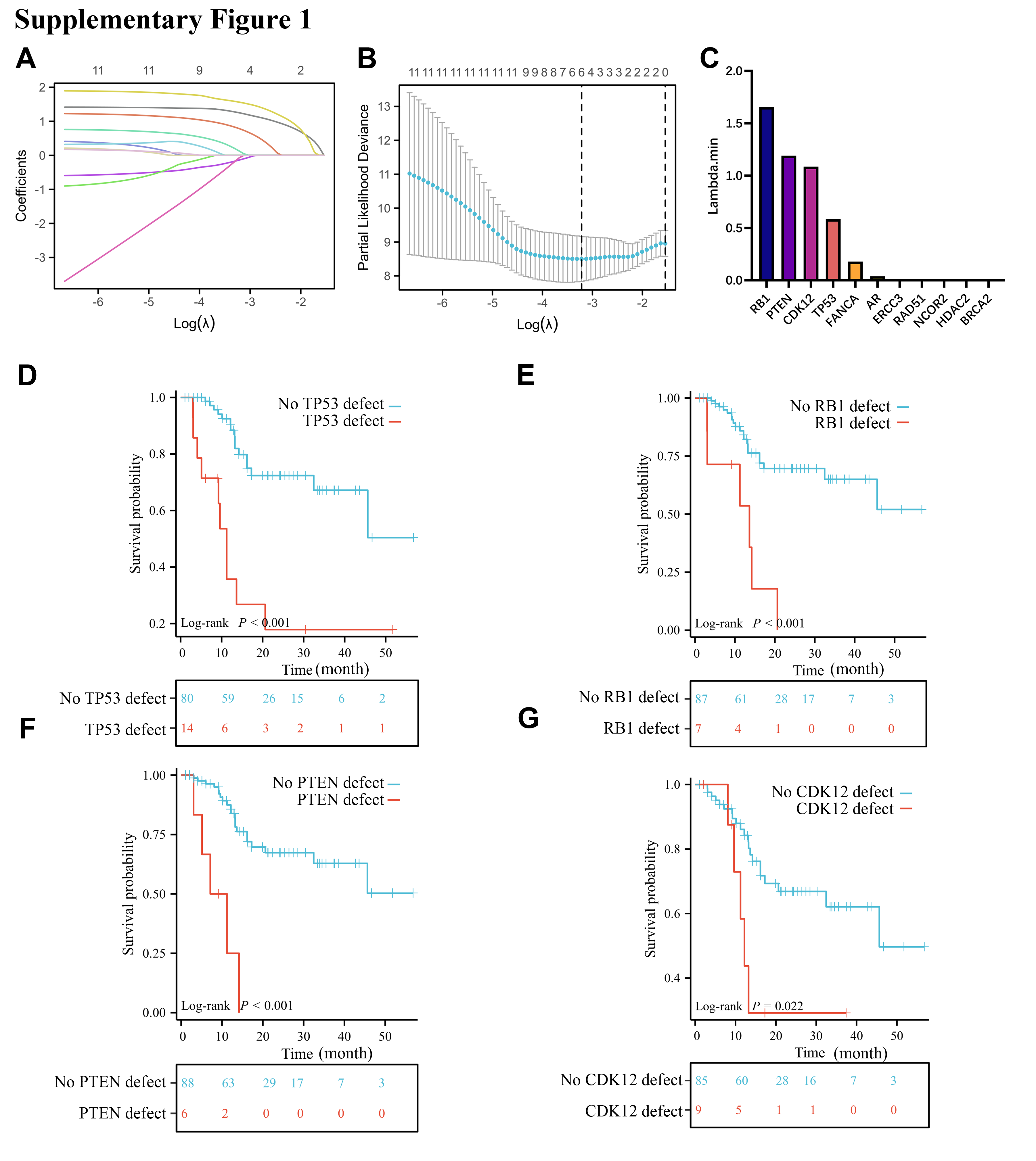

Supplement: Supplementary Figure S1 — Figure S1. Construction of the alternative genes signature for prognostic risk prediction. [file crc-25-0384_supplementary_figure_s1_suppsf1.png]

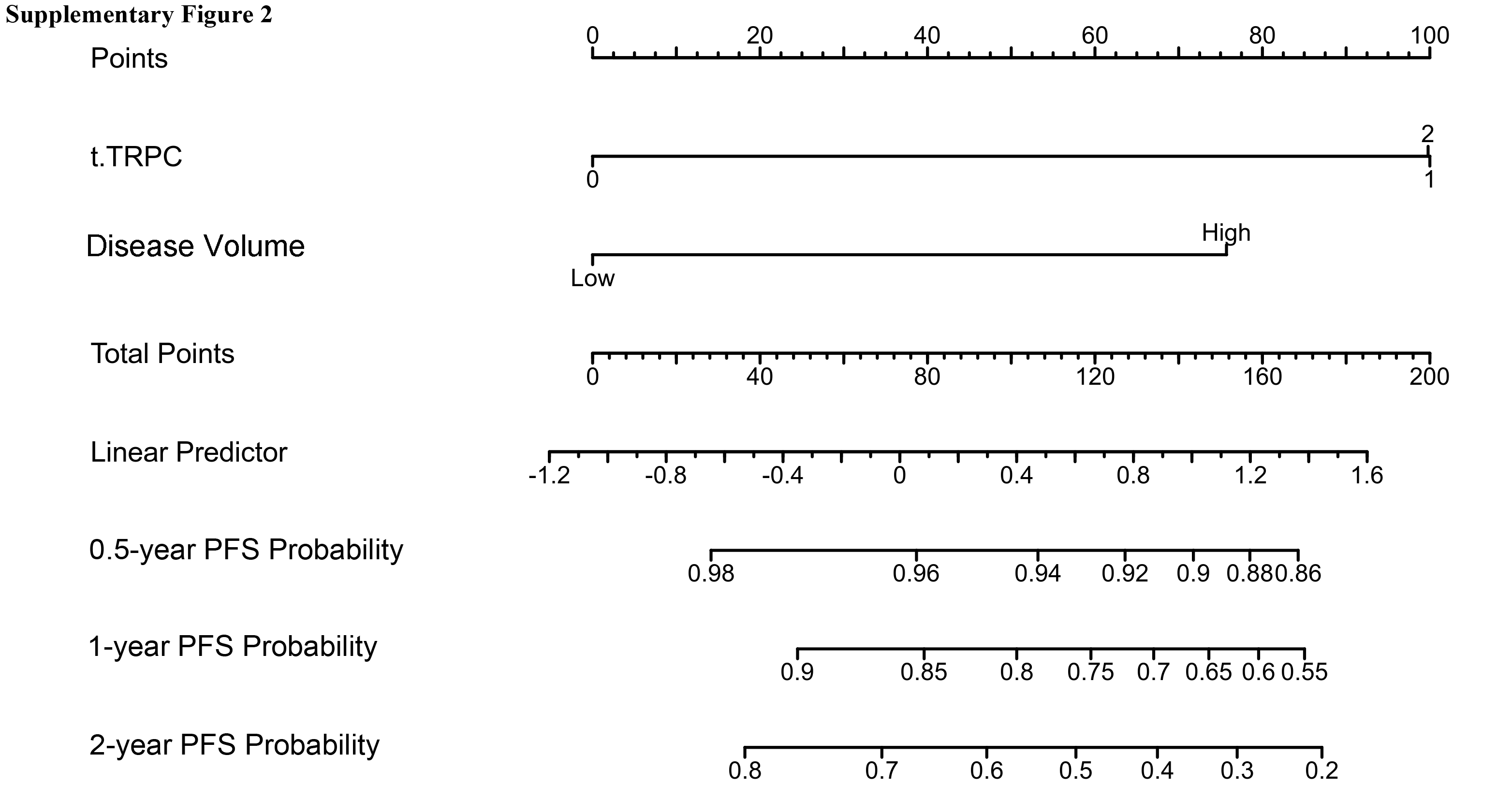

Supplement: Supplementary Figure S2 — Figure S2. Nomogram for predicting progression-free survival (PFS) based on t.TRPC and disease volume score. [file crc-25-0384_supplementary_figure_s2_suppsf2.png]

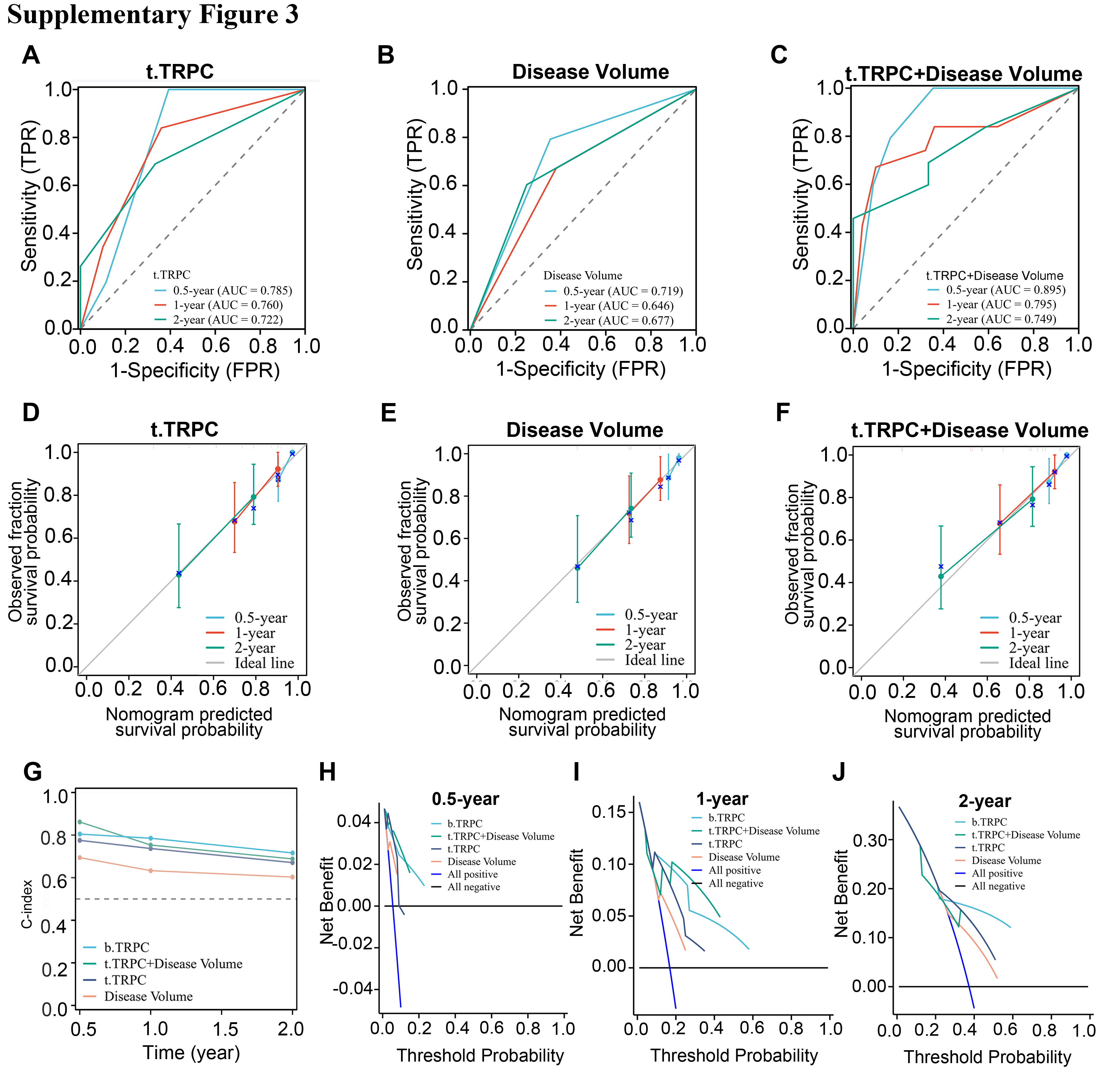

Supplement: Supplementary Figure S3 — Figure S3. Internal validation and clinical utility of the t.TRPC-based prognostic model. [file crc-25-0384_supplementary_figure_s3_suppfs3.png]

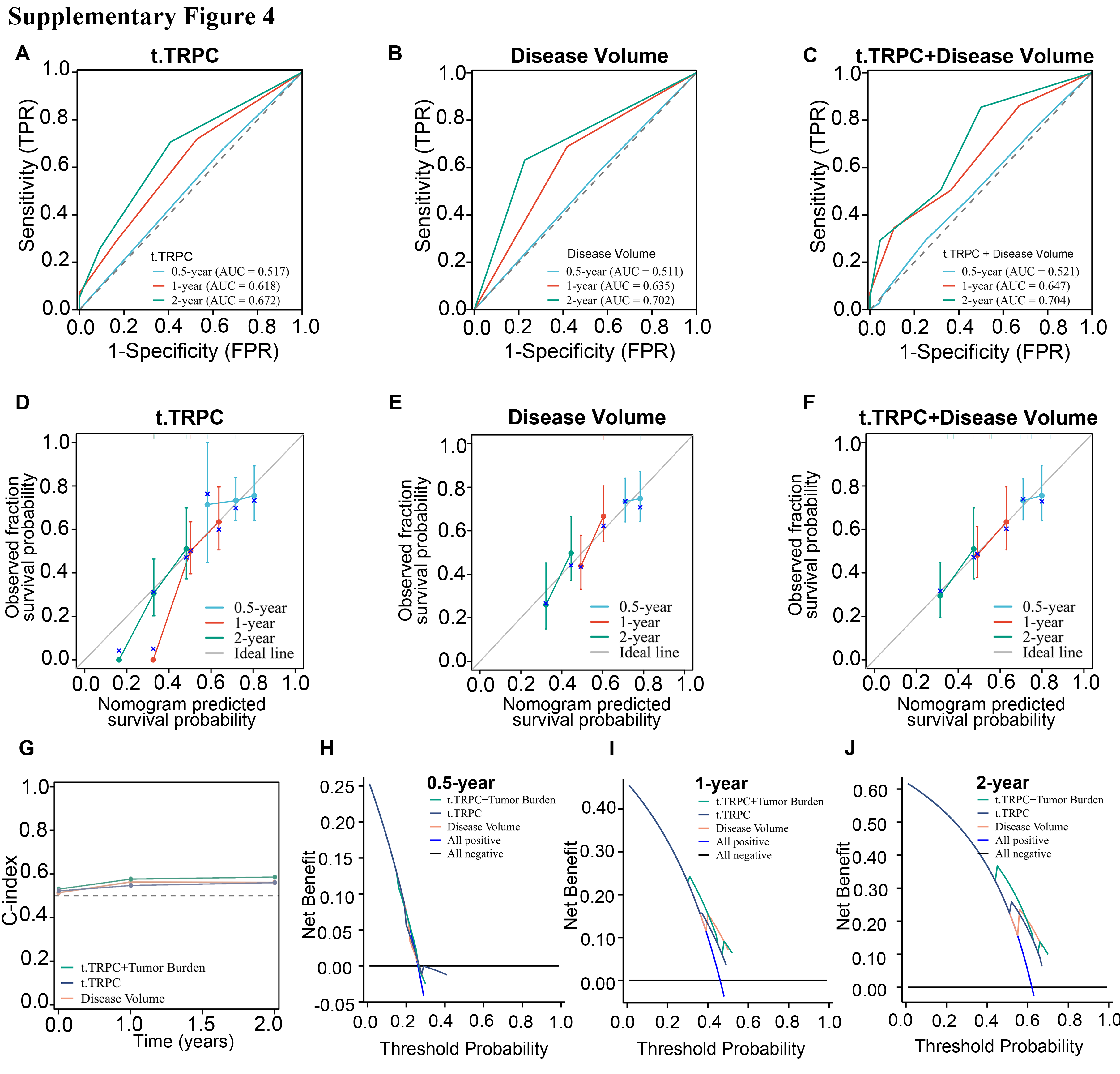

Supplement: Supplementary Figure S4 — Figure S4. External validation and clinical applicability of the t.TRPC-based prognostic model in the validation cohort. [file crc-25-0384_supplementary_figure_s4_suppsf4.png]
